# Supplementary material for: Detection of virus-neutralising antibodies and associated factors against rabies in the vaccinated household dogs of Kathmandu Valley, Nepal
Source: PLoS One. 2020 Apr 27;15(4):e0231967. doi: 10.1371/journal.pone.0231967 (PMC7185695; doi:10.1371/journal.pone.0231967)

**Optical densities values for ELISA plate 1 and 2**

**(Data processed with Rabies-QT-ELISA-BIORAD-Vers.201610.K.XLS)**

a. Optical densities values for plate 1


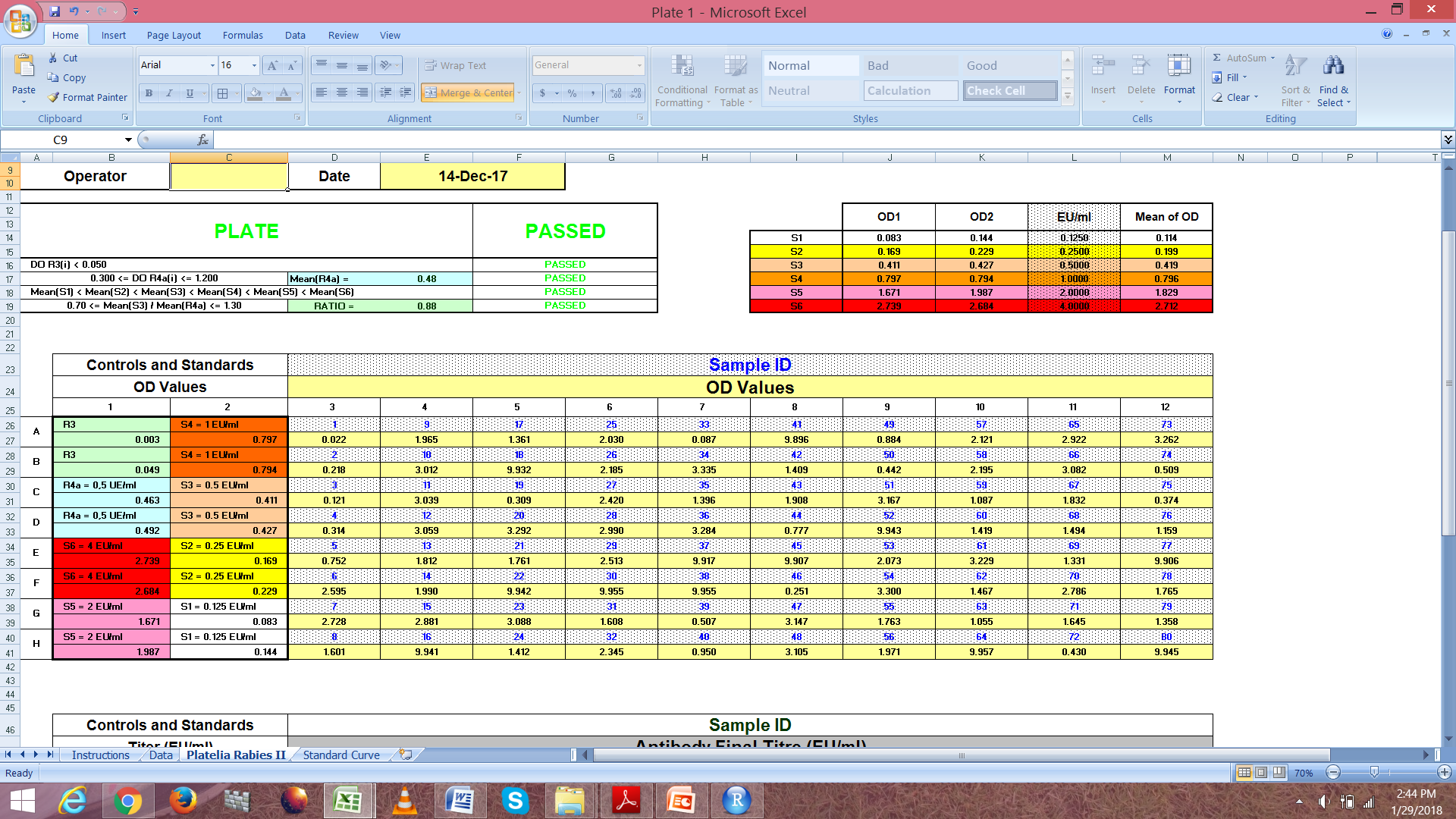


b. Optical densities values for plate 2


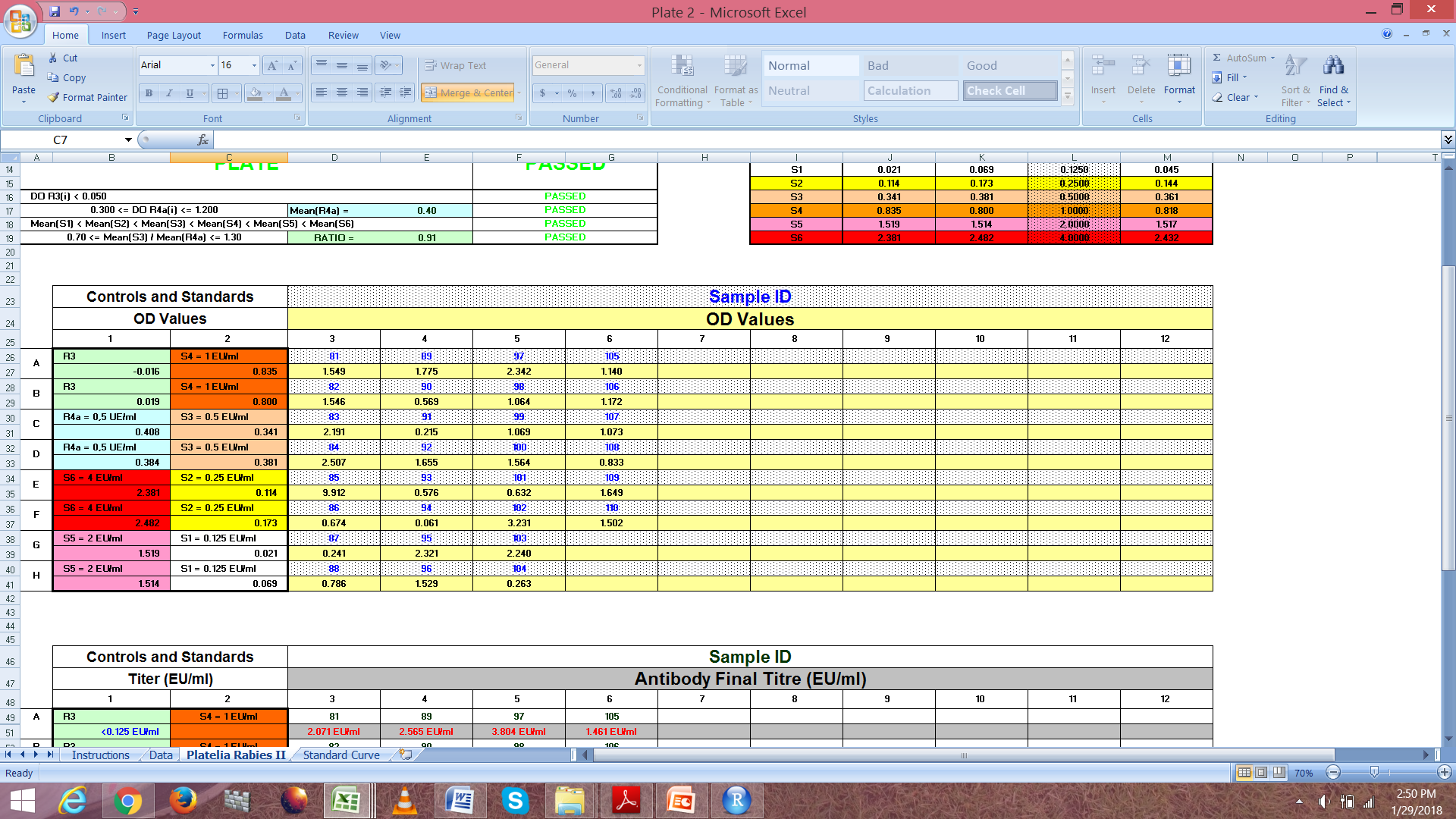

Supplement: S1 File — (DOCX) [file pone.0231967.s002.docx]
