# Supplementary material for: Detection of virus-neutralising antibodies and associated factors against rabies in the vaccinated household dogs of Kathmandu Valley, Nepal
Source: PLoS One. 2020 Apr 27;15(4):e0231967. doi: 10.1371/journal.pone.0231967 (PMC7185695; doi:10.1371/journal.pone.0231967)
Supplement: S3 File — (DOCX) [file pone.0231967.s004.docx]

**Questionnaire used for data collection**

Questionnaire No: ________________________________

Date of the Survey: ________________________________

**A. General Information of Dog Owners**

Name of the Owner: _______________________________

Address: ________________________

Telephone No: ________________________

**B. General Information of Dogs**

a. Name of Dog:

b. Breed:

c. Age:

d. Sex:

1. Is the dog vaccinated against rabies?

🞏 Yes 🞏 No

2. Is this the 1st vaccination for rabies?

🞏 Yes 🞏 No

**If No,** At what age was the dog 1st vaccinated against rabies?

Please specify ______________________________

3. Did the dog get booster vaccination for rabies?

🞏 Yes 🞏 No

**If Yes,** How often did the dog get booster vaccine?

Please specify ______________________________

4. At what age did the dog get his last rabies booster vaccination?

Please specify ______________________________

5. Do you have the vaccination record?

🞏 Yes 🞏 No

6. Was the dog healthy at every rabies vaccination?

🞏 Yes 🞏 No

If No, please specify: ____________________________

7. Did the dog show any rashes/ allergy after rabies vaccination?

🞏 Yes 🞏 No 🞏 Others

If others, please specify: ____________________________

8. Where did you vaccinate the dog?

🞏 Home 🞏 Clinic 🞏 Kennel Club

9. Who gave the rabies vaccination?

🞏 Vet 🞏 Para Vet 🞏 Others

If others, please specify: _______________________________

10. From where was the rabies vaccine taken out for vaccination?

🞏 Fridge 🞏 Ice box 🞏 Others

If others, please specify: _______________________________

11. Was the dog given any other vaccine together with rabies vaccine?

🞏 Yes 🞏 No

**If Yes,** please specify: ____________________________

12. Which vaccine was used during rabies vaccination?

🞏 Vaccine A 🞏 Vaccine B 🞏 Vaccine C 🞏 Others

If others, please specify: _______________________________

13. Where is the dog kept?

🞏 Inside House 🞏 Outside House

14. How is the dog kept?

🞏 Tied 🞏 Free 🞏 Others

If others, please specify: _______________________________

15. How many dogs do you have?

Please specify: _______________________________

16. Do you have any other pets in your house?

🞏 Yes 🞏 No

**If Yes,** please specify: ____________________________

**AND,** Do the pets stay together?

🞏 Yes 🞏 No

17. Does the dog come in contact with stray dogs?

🞏 Yes 🞏 No

**If Yes,** please specify how: ____________________________

18. What kind of feed to you give to the dog?

🞏 Dog Food 🞏 Home Prepared Food 🞏 Others

If others, please specify: _______________________________

19. Do you know about Rabies disease?

🞏 Yes 🞏 No

20. Do you think Rabies is a fatal disease?

🞏 Yes 🞏 No

21. Do you know that Rabies is transmitted from animals to humans?

🞏 Yes 🞏 No

22. Have you ever heard about any human/animal rabies case in your area?

🞏 Yes 🞏 No

23. Have you ever been bitten by a dog?

🞏 Yes 🞏 No

**If Yes,** What did you do after that?

Please specify: ___________________________

24. Has your dog bitten any of the family members?

🞏 Yes 🞏 No

**If Yes,** please specify how many: ___________________________

25. Has your dog bitten any other person except family member?

🞏 Yes 🞏 No

**If Yes,** please specify how many: ___________________________

26. Has your dog been bitten by any stray dogs?

🞏 Yes 🞏 No

27. Have you provided any kind of training to the dog?

🞏 Yes 🞏 No

**If Yes,** Who gave the training to the dog?

🞏 Trainer 🞏 Yourself 🞏 Others

**If others,** please specify: ______________________
