# Supplementary material for: Detection of virus-neutralising antibodies and associated factors against rabies in the vaccinated household dogs of Kathmandu Valley, Nepal
Source: PLoS One. 2020 Apr 27;15(4):e0231967. doi: 10.1371/journal.pone.0231967 (PMC7185695; doi:10.1371/journal.pone.0231967)
Supplement: S4 File — (DOCX) [file pone.0231967.s005.docx]

**Consent form for blood collection**

**Project Title:** Factors Associated with Rabies Antibody Titer of Vaccinated Dogs in Kathmandu Valley, Nepal

**Principal Investigator:** Shikha Rimal, MVPH Student, Chiang Mai University

PURPOSE OF THE FORM:

You are being asked to give your consent to have your Dog participate in a research study by allowing to draw blood from the dog. This consent form gives you the information you will need to help you decide whether to allow your animal to participate. Please read the form carefully. You may ask any questions about the research, the possible risks and benefits, rights as a volunteer participant, and anything else that is not clear. When all of your questions have been answered, you can decide if you will allow your animal to be in this study or not.

WHAT WILL HAPPEN: Blood samples will be collected from Dogs causing minimal injury and pain to the dog.

RISKS OF THE PROCEDURE: In rare cases, formation of Hematoma

IS THERE COMPENSATION FOR PARTICIPATING: No compensation for participation

ARE THERE ANY COSTS FOR PARTICIPATING: No cost involved

CONSENT FOR PROCEDURE:

Your participation in this study is entirely voluntary and you may say No to it. Your signature indicates that this research procedure has been explained to you, that your questions have been answered and that you agree to allow your animal to be a part of this study.

Animal’s Name:_______________________________

Owner’s Name:_____________________________

Owner’s Signature: _________________________________

Date: ________________________________

Name of the Animal Clinic/ Hospital:________________________________

Owner’s Name:________________________________

Pet’s Name: ________________________________

Phone Number: ________________________________

Your pet has been selected for a blood collection procedure for a research today. Please be assured that the researcher, doctors and staff at the Animal Clinic/Hospital will use the safest way available during the blood collection procedure with minimal pain inflicted to the animal. The procedure, however, will have a slight element of risk to the animal. The risk, which is a rare one, can be formation of hematomas at the collection site.

**Owner’s Consent:**

I have read and fully understand this consent form. I understand that I should not sign this form if all items, including my questions, have not been explained or answered to my satisfaction. Or if I do not understand the terms or wording contained in this consent form.

Signature:_________________________

Date:__________________
