# Supplementary material for: Detection of virus-neutralising antibodies and associated factors against rabies in the vaccinated household dogs of Kathmandu Valley, Nepal
Source: PLoS One. 2020 Apr 27;15(4):e0231967. doi: 10.1371/journal.pone.0231967 (PMC7185695; doi:10.1371/journal.pone.0231967)
Supplement: S5 File — (DOCX) [file pone.0231967.s006.docx]

**Consent form translated to local language (Nepali)**

सहमति फारम

परियोजना शीर्षक: नेपालको काठमाडौ उपत्यकामा, खोप लगाको कुकुरहरूको रेबिज एंटीबॉडी टाइटरसँग सम्बन्धित कारकहरू

प्रिंसिपल अन्वेषक: शिखा रिमाल, मास्टर्स भेटनरी पब्लिक हेल्थको बिद्यार्थी, चियांग माई बिश्वो विद्यालय

फारम पछाडिको कारण:

तपाईसंग तपाईको कुकुरलाई यो अनुसन्धानमा सहभागी गराउन र अनुसन्धानको लागि कुकुरबाट रगत तान्न अनुमति मागिएको हो । यो सहमति फारमले तपाईंलाई जानकारी दिन्छ जसबाट तपाइलाई आफ्नो कुकुरलाई यो अनुसन्धानमा भाग लिन दिने या नदिने भन्ने निर्णय लिन मद्दत पुर्याउछ । फारम ध्यानपूर्वक पढ्नुहोस्। तपाई अनुसन्धानको बारेमा, त्येसका सम्भावित जोखिम र फाईदाहरू, एक स्वैच्छिक सहभागीको अधिकार र अन्य स्पष्ट नभएका कुरा कुनै छन् भने प्रश्न सोध्न सक्नुहुनेछ। जब तपाईंका सबै प्रश्नहरूका जवाफ पाउनु भएमा, तपाईं निर्णय गर्न सक्नुहुनेछ कि तपाइँ आफ्नो जनावरलाई यस अध्ययनमा हुने अनुमति दिनुहुन्छ वा दिनुहुन्न।

के हुनेछ: कुकुरबाट रगत झिकिनेछ कम चोट र दुखाइ दिएर

प्रोसेसको जोखिम: दुर्लभ मामलाहरुमा हेमेटोमा हुनसक्छ

सहभागीको लागि क्षतिपूर्ति: सहभागीको लागी कुनै क्षतिपूर्ति छैन

सहभागीको लागि लागत: कुनै पनि लागत छैन

**प्रोसेसको लागि सहमति:**

यस अध्ययनमा तपाईंको सहभागी पूर्णतया स्वैच्छिक छ र तपाई यसको लागि नाई भन्न सक्नुहुन्छ । तपाईंको हस्ताक्षरले संकेत गर्दछ कि यो अनुसन्धान प्रक्रिया तपाईलाई व्याख्या गरिएको छ, तपाइँका प्रश्नहरू जवाफ दिइएका छन् र तपाइँ आफ्नो जनावर यो अध्ययनमा सहभागी हुन अनुमति दिनुहुन्छ । साथै अध्ययनमा संकलन गर्न खोजिएको जानकारीको लागि तयार पारिएको प्रश्नावली फारममा सोधिएको प्रश्नको उत्तर दिएर सहयोग गर्नुहुन्छ ।

पशुको नाम: ________________________

मालिकको नाम: ___________________________

मालिकको हस्ताक्षर: _____________________

मिति: ________________________________

सहमति फारम

पशु क्लिनिक / अस्पतालको नाम: ________________________________

मालिकको नाम: ________________________________

 पेटको नाम: ________________________________

फोन नम्बर: ________________________________

आजको अनुसन्धानको लागी तपाईंको कुकुर रगत संग्रह प्रक्रियाको लागि चयन गरिएको छ। कृपया आश्वासन पाउनुहोस् कि पशु क्लिनिक / अस्पतालमा शोधकर्ता, डाक्टर र कर्मचारीले रक्त संग्रह प्रक्रियाको क्रममा उपलब्ध सबै भन्दा राम्रो तरिकाले जनावरलाई नियन्त्रणमा लिएको, कम दुखाइसंग प्रयोग गर्नेछ। तथापि, प्रक्रियामा पशुको जोखिमको एकदम सानो तत्व हुनेछ। जोखिम, जुन दुर्लभ हो, दुर्लभ मामलाहरुमा हेमेटोमा हुन सक्छ ।

मालिकको सहमति:

मैले फारम पढेको छु र यस सहमति फारम लाई पूर्णतया बुझ्दछु। मैले बुझें कि म यस फारममा हस्ताक्षर गर्नु हुन्न यदि मेरो प्रश्नहरु सहित सबै वस्तुहरु को व्याख्या गरिएको छैन या मेरो संतुष्टि को उत्तर दिएको छैन । वा यदि मैले यो सहमति फारममा निहित नियम वा शब्द बुझ्न सकेन भने। अध्ययनमा संकलन गर्न खोजिएको जानकारीको लागि तयार पारिएको प्रश्नावली फारममा सोधिएको प्रश्नको उत्तर दिएर सहयोग पनि गर्नेछु ।

हस्ताक्षर: _________________________

मिति: __________________
