# Supplementary figures and images for: Detection of virus-neutralising antibodies and associated factors against rabies in the vaccinated household dogs of Kathmandu Valley, Nepal
Source: PLoS One. 2020 Apr 27;15(4):e0231967. doi: 10.1371/journal.pone.0231967 (PMC7185695; doi:10.1371/journal.pone.0231967)

**Animal Ethical Clearance Letter**


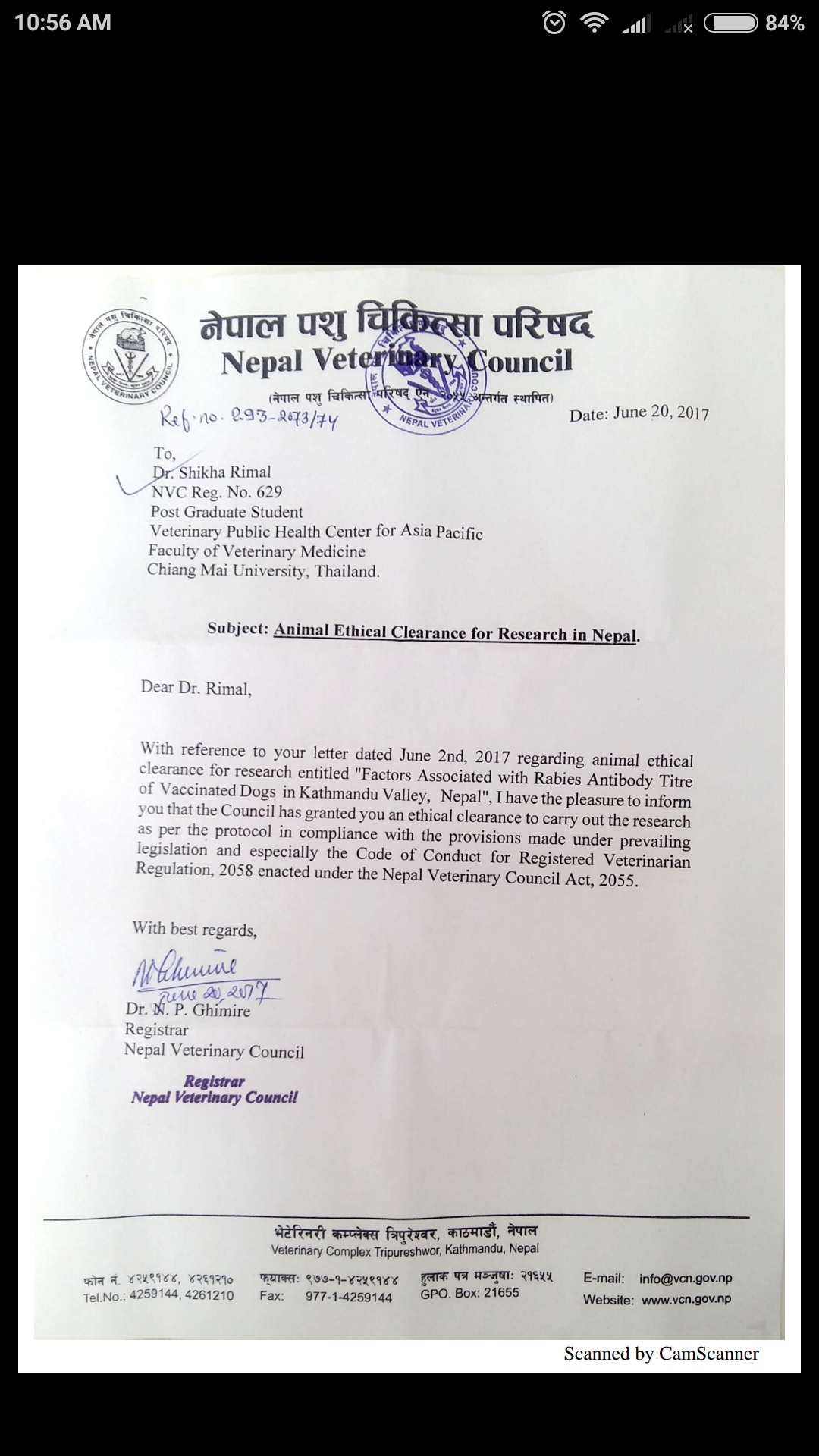

Supplement: S6 File — (DOCX) [file pone.0231967.s007.docx]

**Human Ethical Clearance Letter**


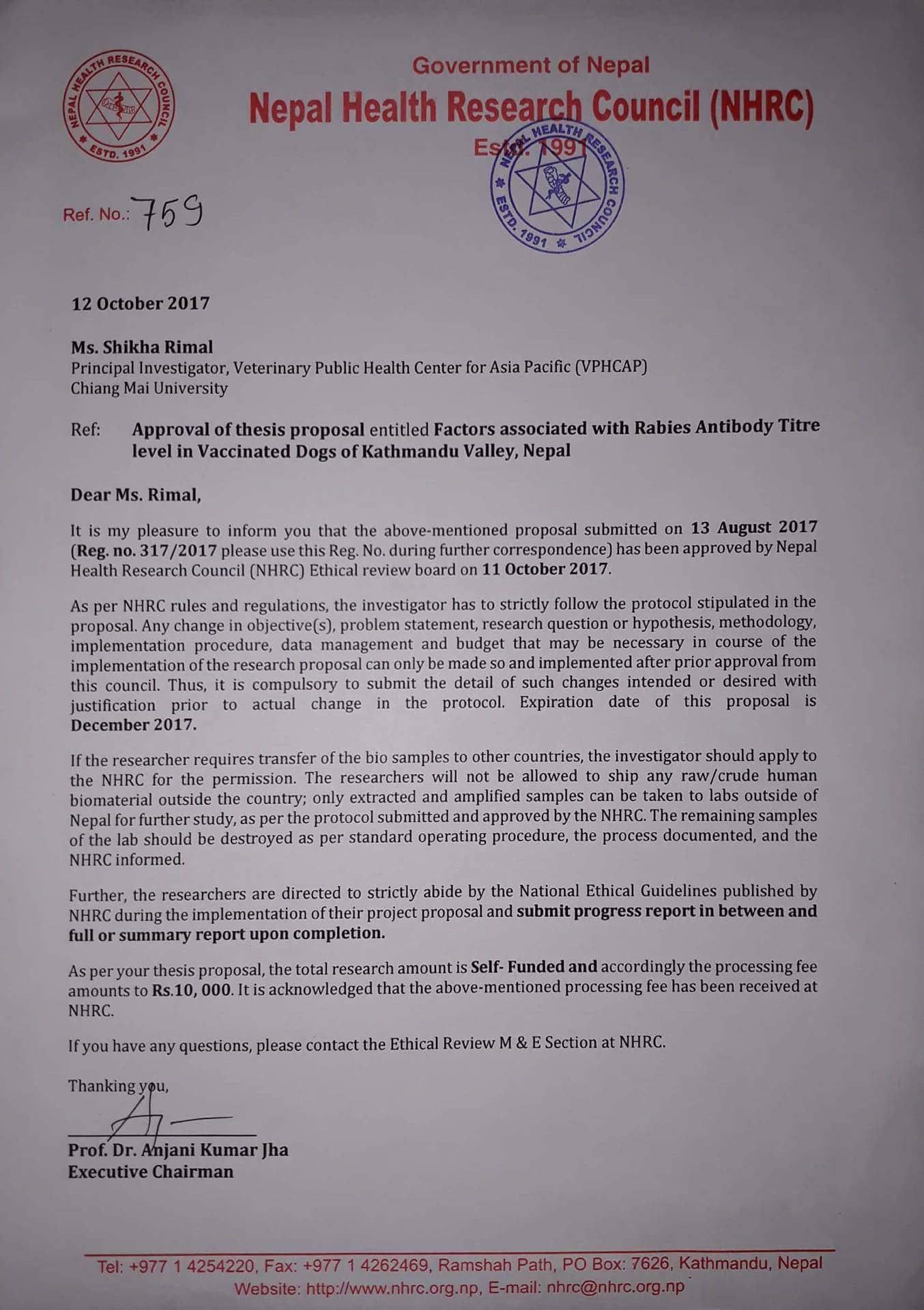

Supplement: S7 File — (DOCX) [file pone.0231967.s008.docx]
