# Supplementary material for: Detection of virus-neutralising antibodies and associated factors against rabies in the vaccinated household dogs of Kathmandu Valley, Nepal
Source: PLoS One. 2020 Apr 27;15(4):e0231967. doi: 10.1371/journal.pone.0231967 (PMC7185695; doi:10.1371/journal.pone.0231967)

**Certificate of approval for use of laboratory animals at Laboratory Animal Center**


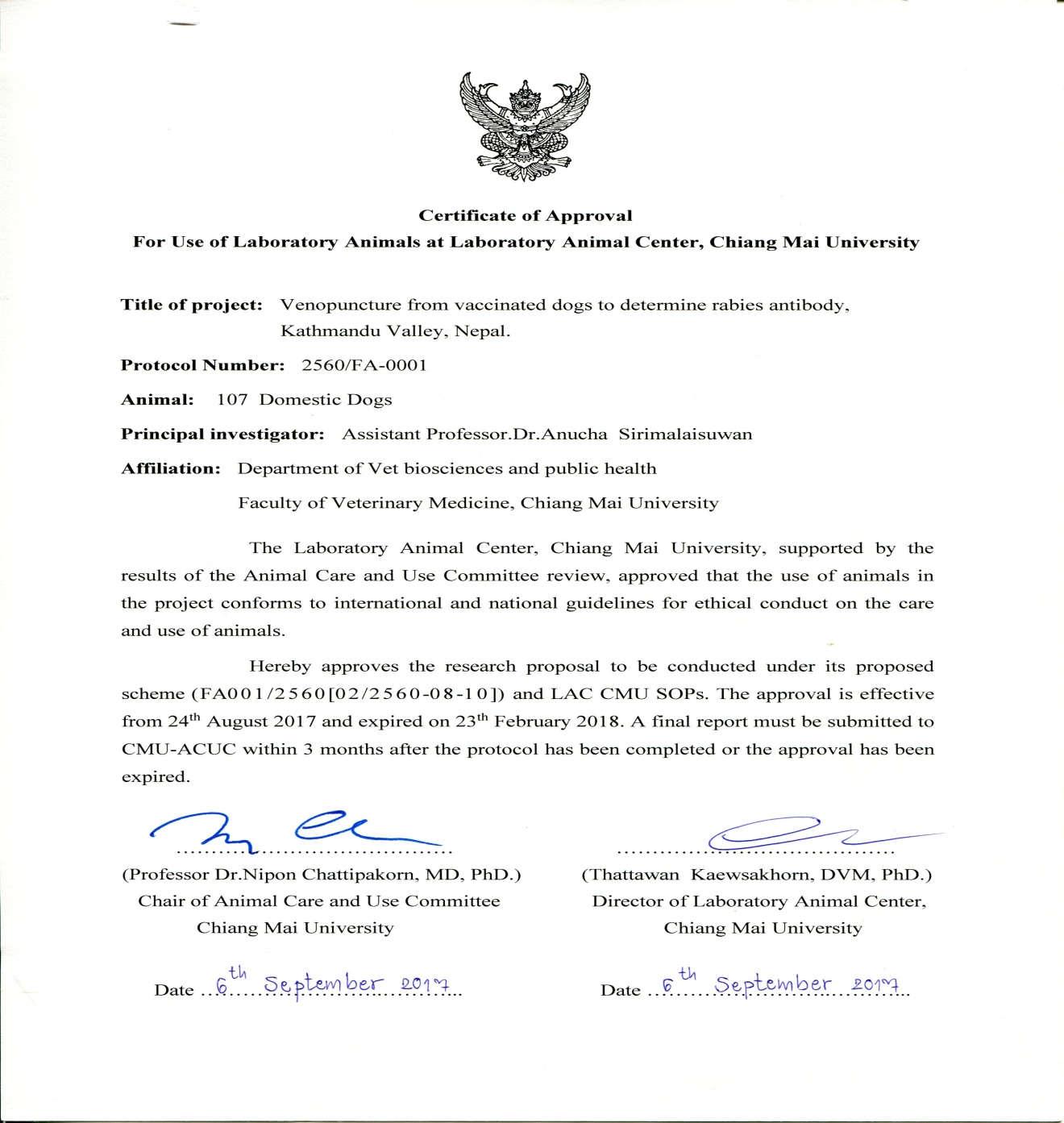


**Certificate of approval for use of laboratory animals at Laboratory Animal Center**


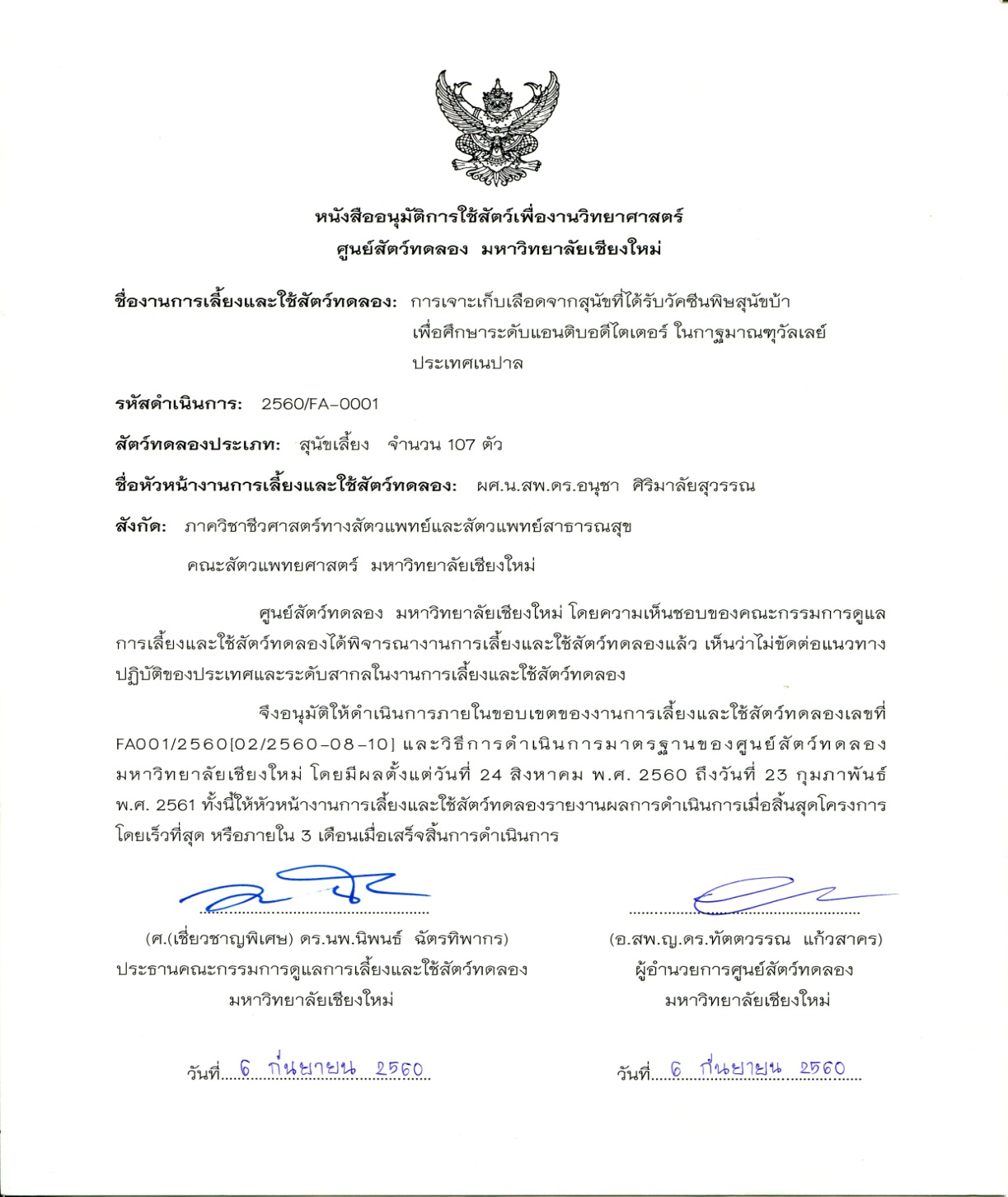

Supplement: S8 File — (DOCX) [file pone.0231967.s009.docx]
