# Supplementary material for: Detection of virus-neutralising antibodies and associated factors against rabies in the vaccinated household dogs of Kathmandu Valley, Nepal
Source: PLoS One. 2020 Apr 27;15(4):e0231967. doi: 10.1371/journal.pone.0231967 (PMC7185695; doi:10.1371/journal.pone.0231967)
Supplement: Attachment — Submitted filename: Comments.docx [file pone.0231967.s010.docx]

**Comments to authors**

Lines 17 and 19: In line 17, you have mentioned rabies impacts heavily on rural but in line 19, there is mention of dogs being the principal vector for rabies transmission in the urban areas. There is a disconnect in the flow of information. Could you please make it clearer. Also, how rabies impacts heavily in the rural and what is the principal vector for rabies in the rural area? Same goes for lines 45 and 46 under author summary.

Line 21: Here, you have mentioned that the study aimed to measure the efficacy of vaccination …….. but the title of this article is “*Factors Associated with Rabies Antibody Titer Levels of Vaccinated Dogs…….”*. to me, the aim of the study and title do not match. In the aim, it seems that to evaluate vaccine efficacy was your primary investigation while the factors to be secondary. However, study title indicates factors for vaccine efficacy to be your primary investigation.

Lines 55 and 56: Please include references.

Line 68 and 69: Please clarify and elaborate as to what is meant by “true status”.

Lines 74, 75 and 76: High rabies cases in humans in Nepal is due to more stray and community dogs or due to more rabies in the pets? Please clarify.

What is the overall prevalence of rabies or prevalence in pet, stray and community dogs? If you have this data then the burden of human rabies due to dogs can be explained.

Lines 78, 79 and 80: could you please rephrase the sentence to make the point clear.

Lines 90 and 91: Please clarify your aim of the study and the title of the study. Your title says factors associated …… but your says different.

Lines 92 and 93: Are the pet dogs vaccinated as a campaign program or as and when requested by the owner?

Line 218 and 219: Here, it is mentioned that the insufficient titres could be due to recent vaccination. But I understand from the inclusion criteria that the blood collection was done one month after the last booster. It would be clear if you had data on the number of dogs that were vaccinated recently. In addition, you could also look for information on the non-respondents to vaccination as some animals innately do not respond irrespective of time since vaccination and type of vaccines.

Lines 228-230: Please add references.

Lines 231-244: The paragraph is informative but does not quite connect with the present study.
