# Supplementary material for: Detection of virus-neutralising antibodies and associated factors against rabies in the vaccinated household dogs of Kathmandu Valley, Nepal
Source: PLoS One. 2020 Apr 27;15(4):e0231967. doi: 10.1371/journal.pone.0231967 (PMC7185695; doi:10.1371/journal.pone.0231967)
Supplement: Attachment — Submitted filename: Response of reviewers comments 20200312.docx [file pone.0231967.s011.docx]

**Comments to authors**

Lines 17 and 19: In line 17, you have mentioned rabies impacts heavily on rural but in line 19, there is mention of dogs being the principal vector for rabies transmission in the urban areas. There is a disconnect in the flow of information. Could you please make it clearer. Also, how rabies impacts heavily in the rural and what is the principal vector for rabies in the rural area? Same goes for lines 45 and 46 under author summary.

**Response: We appreciate your comments. To make the sentences more coherence and follow information, we have modified the sentences in lines 17, 19, 45 and 46 which are highlighted by red color for your kind information.**

Line 21: Here, you have mentioned that the study aimed to measure the efficacy of vaccination …….. but the title of this article is “*Factors Associated with Rabies Antibody Titer Levels of Vaccinated Dogs…….”*. to me, the aim of the study and title do not match. In the aim, it seems that to evaluate vaccine efficacy was your primary investigation while the factors to be secondary. However, study title indicates factors for vaccine efficacy to be your primary investigation.

**Response: Thank you so much for your comments. We have changed title and aim of study as per your suggestions.**

Lines 55 and 56: Please include references.

**Response: As per your suggestions, we have incorporated reference in lines 55 and 56 that is highlighted by red color.**

Line 68 and 69: Please clarify and elaborate as to what is meant by “true status”.

**Response: Thank you for your comment “ true status” meant rabies burden in Nepal which has been corrected in the lines 68 and 69.**

Lines 74, 75 and 76: High rabies cases in humans in Nepal is due to more stray and community dogs or due to more rabies in the pets? Please clarify.

What is the overall prevalence of rabies or prevalence in pet, stray and community dogs? If you have this data then the burden of human rabies due to dogs can be explained.

**Response: Thank you for your comments. Host wise prevalence study also suggests that 61.7% rabies cases were detected in stray and community dogs (Pant et al, 2013). Therefore, high human rabies cases are due to stray and community dogs in Nepal. We have added one supporting reference in line 74, 75 and 76.**

Lines 78, 79 and 80: could you please rephrase the sentence to make the point clear.

**Response: As per your suggestions, we rephrased in lines 78, 79 and 80 for your kind information that is indicated by red color.**

Lines 90 and 91: Please clarify your aim of the study and the title of the study. Your title says factors associated …… but your says different.

**Response: Thank you very much for your kind comments. We have corrected it as per your previous suggestions mentioned in line 21.**

Lines 92 and 93: Are the pet dogs vaccinated as a campaign program or as and when requested by the owner?

**Response: Thank you very much for your comments. In Nepal, the pet dogs are vaccinated both in the campaign program as well as requested of dog owners.**

Line 218 and 219: Here, it is mentioned that the insufficient titres could be due to recent vaccination. But I understand from the inclusion criteria that the blood collection was done one month after the last booster. It would be clear if you had data on the number of dogs that were vaccinated recently. In addition, you could also look for information on the non-respondents to vaccination as some animals innately do not respond irrespective of time since vaccination and type of vaccines.

**Response: As per your suggestions, we have incorporated influencing factors for insufficient production of antibody titer level. The factor like age, breeds, type of vaccines could be influencing factor. We have also inserted supporting information and reference.**

Lines 228-230: Please add references.

**Response: As per your suggestions, we have inserted reference number 23 in line 228-230 for your kind information.**

Lines 231-244: The paragraph is informative but does not quite connect with the present study.

**Response: We appreciate your comments and we also realized that the paragraph is more informative but irrelevant in this study so, we removed it from this manuscript.**

Reviewer #1: Overall, the manuscript is good and worth publishing. However, it needs refining and edition to make the information clear. One of the important point to change would be to align the aim of the study and the title of the study.

**Response: Thank you very much for your comments. We have corrected all comments assigned by reviewer 1.**

Reviewer #2: This is a very well written, and interesting article, which I thoroughly enjoyed reading. I have made a few minor comments which are mainly grammatical, but also have a few questions regarding the study and interpretation. Most of the comments are minor. I anticipate being asked to review this again, so if it is a minor grammatical change, please don’t feel that you need to write a big long rebuttal.

Line 21- I am not a fan of using first person words like our in scientific writing. Maybe change this to this study identified….. ?

**Response: Thank you very much for your kind comments. We have changed first personal pronoun “our” into this study for your kind information.**
Line 27- you can remove the word out, as separated is sufficient

**Response: As per your suggestions, we removed “out” from the line 27 that is highlighted by red color.**

Lines 35-37- you talk about seroconversion levels. I do wonder if antibody titre would be better as undetectable seroconversion levels suggests that the animal hasn’t seroconverted?

**Response: Thank you very much for your kind comments. We followed WHO guidelines. According to WHO, the recommended level of protection is (≥ 0.5 IU/ml) but we used (*Platelia^TM^ Rabies II kit ad usum Veterinarium,* Biorad, China*.* This test had 98.6% specificity and 88.8% sensitivity. For detecting antibody titer level there are other gold standard methods like rapid fluorescent focus inhibition test (RFFIT) it can detect seroconversion level of rabies in dogs.**

Line 42- you talk about the Nepalese vaccine, and it almost sounds as if you expect it not to be as good as international vaccines. Is there a reason for that?

**Response: In Nepal, cell culture rabies vaccine was produced by rabies vaccine production laboratory (RVPL) and animal trials were done in 2002. Since then, vaccine efficacy has not been conducted till date. This is our first comparative efficacy trials of rabies vaccination in Nepal. This study will generate base line data for the control and eliminated rabies in Nepal.**

Line 50- a high level of rabies antibody titre sounds strange- please reword

**Response: We have changed high level of rabies antibody titer in line 50 that is highlighted by red color.**

Line 55- comma after preventable, and after neglected will help flow

**Response: We did it.**
Line 58- delete out

**Response: Thank you sir, we deleted out from manuscript.**
Line 61- a semi colon after India may aid flow

**Response: We have put a semi colon after India in text that is highlighted by red color.**
Line 66- a comma after There may aid flow

**Response: We did it for your kind information.**
Line 74- you talk about spill over from stray and community dogs- is there any spill over from wildlife?

**Response: Thank you for your comments. Sylvatic rabies cycle exists in Nepal but human rabies cases spilled from wild life animal has not been reported yet.**

Line 78- you talk about high level vaccination coverage- is this in Nepal or elsewhere? It would be nice to clarify that to make it clearer

**Response: Thank you very much for your comments. It is WHO recommendation and we have modified these sentences as per reviewers 1 comments and inserted reference also for your kind information.**
Line 98- as part of the questionnaire, you ask about recipient of vaccination. In the Uk and US you get a confirmation of vaccination. Is that available in Nepal? And if not, how do you know that they were telling the truth?

**Response: Thank for your comments. Dog owners are provided receipt of vaccination cards of their dogs in Nepal. Before blood sample collection, we had checked vaccination cards in details.**
Line 103- How was the blood separated out?

**Response: We have inserted blood separation method in methodology parts.**
Line 109- you mention the specificity and sensitivity of the assay- is this done as part of the study or is it what the manufacturer has recommended? Please state that in the manuscript

**Response: The manufacturers company has mentioned specificity and sensitivity in the kits 98.6% specificity and 88.8% sensitivity. We have also mentioned its sensitivity and specificity in manuscript as per you suggestions.**

Line 110- sample dilution- how was this done? In what? And to what concentration?

**Response: Thank you very much. The sample dilution was done 1/10 ratio (10 μl of sample in 990 μl of dilution solution).**
Line 112- 3 needs to be in full, and it would be three washing steps (rather than washings)

**Response: We have corrected it.**
Line 114- 5 needs to be in full, and it would read better as five washing steps

**Response: We have corrected it.**
Line 115- comma after well

**Response: We have corrected it.**

Line17- what is 1N- should this be 1M for molarity?

**Response: 1 N mean Normal solution of sulphuric acid (H2SO4) which was provide in test kit.**

Line 149- required may be better than requested

**Response: We have changed it.**
Line 149-151- again seroconversion may be better written as antibody titres?

**Response: We have corrected it.**

Line 154- It would be very good to name the vaccines in here as the company, and the people would benefit from knowing from this relative lack of efficacy.
You also talk about three different types of vaccine- are they different makes, killed, modified live? A bit more detail in here would be useful

**Response: We have mentioned vaccine details with company name in manuscript.**
Line 155-156- this reads a bit unclearly, but I cannot work out a better way to put it

**Response: We have changed it as per your suggestions.**
Line 159- you discuss vaccine failures, but I think to call it a failure is difficult due to so many potential reasons why they may not work. Poor administration, out of date, poor storage etc. Maybe tone this down, or discuss reasons why they may have failed

**Response: Thank you very much for your comments. We have discussed the influencing factors associated with vaccine failures in manuscript.**
Line 161- 7 in words

**Response: We have corrected it.**
Line 162-163- it is reported that black and tan dogs respond poorly, particularly to parvovirus vaccines. Is there any evidence of this here?

**Response: This is not any evidence of poor vaccine efficacy of parvovirus reported in Nepal.**
Line 170- you analyse 28 different factors but don’t mention which ones. It would be good to have some idea of what you analysed

**Response: Thank you for the comments. We have analyzed 28 different factors which are described in the methodology parts. Only four influencing factors were associated with antibody titer and these are mentioned in the analysis table.**
Line 174- again, We and our would be better replaced with non first person words

**Response: We have corrected it.**
Line 179- can remove the P. from P. Singh

**Response: We have corrected it.**
Line 179 … showed only 16% of household ….

**Response: We have corrected it.**
Lines 177-183- you discuss different studies which have different antibody titres. How many of these are vaccine induced? Or doesn’t it say?

**Response: Thank you for your comments. Details vaccination status and administrated doses has not clearly mentioned in that reference paper.**

Line 186- 3 in words

**Response: We have corrected it.**
Line 189- as stated above, I feel calling it a vaccine failure is a bit misleading, and maybe need quantifying.

**Response: Thank you. We have corrected it Sir.**
Line 193-196- a bit more detail on the vaccines and how they differ maybe useful here- strains, administration, DOI, boosters, preparation etc

**Response:** Three of the most popular vaccines such as Defensor (Killed Virus), Biocan R (Inactivated Strain) and NeJa Rab (Inactivated Cell culture rabies Vaccine) were included in the research. All of the vaccines are administered subcutaneously that have 1 ml capacity vial. Yearly booster of the vaccine is given.

Line 197- 2 should be in words

**Response: We have corrected it.**
Line 197- How much of the vaccine failures could be associated with MDA? Could this be a cause of poor vaccine response in dogs under 2 years?

**Response: Only one dog included in the study was four months of age, the vaccine failure of which might be associated with MDA.**
Line 202- again, replace we

**Response: We have corrected it.**
Line 208- But in this study, no relationship ….

**Response: We have corrected it.**
Line 212- again, replace we

**Response: We have corrected it.**
Line 213- I have not encountered a coronavirus vaccine before? Is this common in Nepal? Is parvovirus not commonly vaccinated against?

**Response: The core vaccination of pets in Kathmandu valley comprises of Anti Rabies Vaccine and Canine Distemper combined vaccine (DHPPiL) which includes Canine Distemper, Canine Hepatitis, Parvo Virus Vaccine, Para Influenza Vaccine and Leptospira Vaccine. Parvo virus vaccination is included in the core vaccination. Corona Virus vaccination is an optional vaccination.**

Line 216 this reads a bit unclearly, consider rewording.

**Response: We have corrected it.**
Line 218- 2 needs to be in words

**Response: We have corrected it.**
Line 219- titre testing. Is there commonly done post vaccination in Nepal? Maybe this could be a suggestion for the conclusions?

**Response: Titer testing is not commonly done after post vaccination in Nepal. We have recommended it in our conclusion part.**

Line 220- again replace we

**Response: We have corrected it.**
Line 222- outdoor dogs would also have chance to interact with wildlife. Is this considered a risk for infection for these animals?

**Response: Dogs that were not kept inside the home had a greater chance to interact with stray dogs and wild life that could be risk of infection for other animals as well as humans.**

Line 227-230- you discuss a low IgG response in some animals. How many animals is this issue thought to affect? Could it be a cause of your poor vaccine response?

**Response: Poor vaccine response had reported in twelve dogs in this study it could be cause due to low IgG titer level.**
Line 237- decreased may sound better than less?

**Response: We have removed this paragraph according to previous reviewer comments.**

Line 239- exposure instead of exposer

**Response: According to first reviewers suggestion, we removed this paragraph from 231-244.**
Line 248- again, replace we

**Response: We have corrected it.**
Line 249- this seems a strange comment considering that you have said that vaccine B gives a poor response?

**Response: We have corrected it.**

Line 251- does anti rabies vaccination need capitals?

**Response: Generally free rabies vaccination program conducted by government, private clinics, kennel club and other NGOs do not charge for this vaccine in the world rabies day (September 28 of each year). Other than that, private clinic and government hospital charge for anti rabies vaccination in regular vaccination schedule.**

Line 252- the alteration from bi-yearly to yearly vaccination is an interesting idea. How does this work with costs? And regulatory bodies?

**Response: Usually a yearly booster vaccine is given. At places, local technicians tend to give a booster vaccination every six months. The cost for the pet owners is more as they pay double the money for the same vaccine. We do not have a regulatory body that monitors this malpractice.**

Line 253- how can yearly vaccination be as economically viable as bi-yearly? You need 2 vaccines instead of one which will cost more?
**Response: Yearly vaccination is more cost effective than bi-yearly. The cost of single dose vaccine is less by half than two doses of the same vaccine.**

Line 257- again, replace we

**Response: We have corrected it.**

Line 258- comma after simultaneously
**Response: We did it.**

Line 260 again replace we

**Response: We have corrected it.**
Line 261- you say here that you collected samples from clinics, but the methods implies more that you go out to houses and test. Could you make this clearer in the methods?
**Response: We corrected this sentences linking with methodology part.**

Could you remove some of the figure legends, as they are in 3 or 4 times?

**Response: We have corrected it.**

Figure 1-= a map of where these areas are in Nepal may be useful as my Nepalese geography is terrible. Is there any differences between the areas?

**Response: We have included an area map as per your suggestions.**

Figure 2- it would be good to label which bit means vaccines, and what the titre is, because these values are low and you mention over 120 in the text which doesn’t link to the figure
Table 1- your second line adds up to 89%- where is the other 11% . Seroconverted could be one word too

**Response: We have added as per your suggestions.**

Table 2- a bit more detail in the table legend as to what all the abbreviations means may be helpful too

**Response: We have added as per your suggestions.**
