# Supplementary material for: Detection of virus-neutralising antibodies and associated factors against rabies in the vaccinated household dogs of Kathmandu Valley, Nepal
Source: PLoS One. 2020 Apr 27;15(4):e0231967. doi: 10.1371/journal.pone.0231967 (PMC7185695; doi:10.1371/journal.pone.0231967)
Supplement: Attachment — Submitted filename: Response to reviewer comments 20200331.docx [file pone.0231967.s012.docx]

Comments to authors

Reviewer #1: (No Response)

Reviewer #2: I have reviewed your manuscript to ensure that it is ready to go for publication. I have made a few minor suggestions for grammatical changes which will increase the flow of the reading. It may look like there is a lot, but it will probably only take you 10 minutes maximum.
This will hopefully increase the speed of publication when it gets to the editorial office.

Response: Thank you very much sir for your kind valuable suggestions and comments for grammatical changes to enhance our revised manuscript flow of the reading. As per your kind suggestions, we have modified and made all single inline grammatical changes in revised manuscript with red color according to assigned by reviewer 2 for your kind information

Line 22- …the aim of detecting virus-neutralising…..
Line 36- …(<125 EU/ml) suggesting that the ….
Line 42- …. manufactured brand of vaccine, which is available in Nepal, is potent …
Line 49- …efficacy of vaccination programmes, and to determine whether ….
Line 57- ….is a vaccine preventable, viral zoonosis ….
Line 58- …all human deaths from rabies are caused ….
Line 59- …59,000 people due from this disease …
Line 60- …of which 30-50% are below ….
Line 61- …60% of the total number of cases ….
Line 70- … the year, and dogs are ….
Line 73 -…involves domesticated dogs, and the other is ….
Line 76- Large numbers of human rabies cases …..
Line 82- For the dog rabies mass vaccination program, achieving a ….
Line 89-… vaccines been used, or to determine if the ….
Line 98- …were taken from the Nepal Veterinary ….
Line 102- agriculture doesn’t need to be capitalised
Lines 98-104- this doesn’t need to be in bold
Line 107- ….districts of Kathmandu valley, shown in map….
Line 108- Inclusion criteria were a dog ages above 4 months ….
Line 120- Biorad, China) was used, following the ….
Line 131- adding to each well, a peroxidase substrate …..
Line 135- ….quantification standards (S1 to S6) , obtained by serial ….
Line 137- …titers in quantification tests, obtained after a ….
Line 143- ….regarding each pet dog (age, sex ….
Line 162- …optical density compared to a standard curve ….
Line 173- please put 3 in words
Line 175. However, there was no significant ….
Line 178- …less than two years old (47%) …
Line 193- …antibody was detected in 110 pet dogs….
Line 194- …89.09% had the necessary ….
Line 195- …..(>0.5 IU/ml), which is in line ….
Line 207- …may be due to the age of the vaccinated ….
Line 217- Higher numbers of vaccine failures ….
Line 218- …in younger dogs, because their ….
Line 219- ….has been documented [19], which may ….
Line 222- …analysis, however, no significant relationship….
Line 223- dog and their titer level was found
Line 228- double space between higher and failure (please remove 1)
Line 232- double space between give and core (please remove 1)
Line 232- …which includes the anti-rabies. …
Line 233- Canine distemper doesn’t need capitals
Line 234- diseases from canine distemper to leptospirosis don’t need capitals
It may also be worth mentioning that there is, as yet, no studies on variation in canine parvovirus published from Nepal. This is one of the diseases which I work on so it maybe worth a chat in the future to get some samples and do this study?
Line 235- coronavirus doesn’t need a capital
Line 235- However, there was no….
Line 236 and 237- Anti-rabies, corona, canine and distemper, don’t need capitals
Line 242- ….insufficient titer level, while two dogs ….
Line 251- wildlife is one word
Line 253- Due to some health conditions, prior to the vaccination….
Line 257- …does not produce sufficient, specific IgG….
Line 262- However, in the market, there is a tendency ….
Line 266- …safely said that anti rabies vaccination ….
Line 273- Titre testing post vaccination …..
